# Response to mechanical loading in rat Achilles tendon healing is influenced by the microbiome

**DOI:** 10.1371/journal.pone.0229908

**Published:** 2020-03-10

**Authors:** Franciele Dietrich-Zagonel, Malin Hammerman, Pernilla Eliasson, Per Aspenberg

**Affiliations:** Orthopedics, Department of Biomedical and Clinical Sciences, Division of Surgery, Orthopedics and Oncology, Faculty of Medicine and Health Science, Linköping University, Linköping, Sweden; Queen Mary University of London, UNITED KINGDOM

## Abstract

We have previously shown that changes in the microbiome influence how the healing tendon responds to different treatments. The aim of this study was to investigate if changes in the microbiome influence the response to mechanical loading during tendon healing. 90 Sprague-Dawley rats were used. Specific Opportunist and Pathogen Free (SOPF) rats were co-housed with Specific Pathogen Free (SPF) rats, carrying *Staphylococcus aureus* and other opportunistic microbes. After 6 weeks of co-housing, the SOPF rats were contaminated which was confirmed by *Staphylococcus aureus* growth. Clean SOPF rats were used as controls. The rats were randomized to full loading or partial unloading by Botox injections in their calf muscles followed by complete Achilles tendon transection. Eight days later, the healing tendons were tested mechanically. The results were analysed by a 2-way ANOVA with interaction between loading and contamination on peak force as the primary outcome and there was an interaction for both peak force (p = 0.049) and stiffness (p = 0.033). Furthermore, partial unloading had a profound effect on most outcome variables. In conclusion, the response to mechanical loading during tendon healing is influenced by changes in the microbiome. Studies aiming for clinical relevance should therefore consider the microbiome of laboratory animals.

## Introduction

Tendon healing is dependent on the immune system [1] which has a profound effect on the strength of the tendon callus [2, 3]. The immune system, and particularly T cells, changes in response to alterations in the microbial environment in the gut and skin [4, 5]. To the extent that T cells play a role in tendon healing, microbial changes in the gut can indirectly influence this healing. Recent data show that changes in the gut microbiota can alter the levels of CD4+ and CD3+ T cells within the tendon callus [1]. Furthermore, changes in the microbiome can influence the response to different immunomodulatory treatments, such as local PRP injections or systemic corticosteroid treatment, during tendon healing [1, 3]. To the best of our knowledge, these two studies are the first to confirm a connection between the microbiome and tendon healing outcomes.

Mechanical loading also profoundly influences tendon healing. In a rat Achilles tendon healing model, complete load protection can reduce the strength of the healing tendon by a factor of 5 [6]. The magnitude of loading appears to activate different mechanisms which will ultimately lead to different effects on the mechanical properties [7]. The Achilles tendon heals through callus formation and the initial tissue contains weak matrix with primarily leukocytes infiltrating this matrix [8]. Full loading, when applied to this matrix, has been shown to induce microdamage and alter the immune cell composition and ultimately promote a pro-inflammatory response [7, 9]. Hence, in part, the response to mechanical loading seems to be closely connected to immune cell reactions and thereby possibly to changes in the microbiome [8–10]. We therefore hypothesized that changes in the microbiome, would also influence the response to mechanical loading.

## Results

The SPF rats were only used for co-housing as they came from a different breeder facility. Therefore, only SOPF animals (clean and contaminated) were used for the actual experiment and data collection.

### Co-housing model and contamination procedure

**Fig 1:**
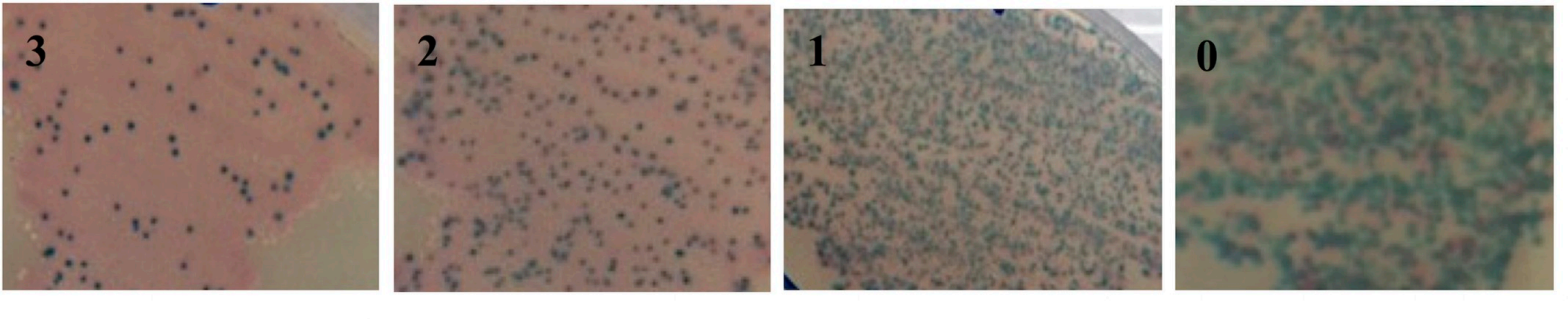
Bacteria growing on CHROMagar Staph Chrom plates, from oral swabs of contaminate rats. The pink colour means growth of *S*. *aureus*. The green colour means growth of other bacteria. Numbers mean: 3 dominant presence of *S*. *aureus*; 2 more than half; 1 means minimal; and 0 means no detected *Staphyloccocus aureus*. Only rats with growth grade 2 and 3 were used for data collection.

22 out of the 30 co-housed SOPF rats showed *Staphyloccocus aureus*, growth grade 2 or more. The remaining 8 rats with little or no growth were excluded from the analysis (Fig 1).

### Microbial community status

6 weeks of co-housing lead to higher levels of *Staphylococcus aureus* and *Escherichia coli* in the gut microbiota of the contaminated SOPF rats compared to the clean SOPF rats, where the levels of these bacteria were below the detectable limits. However, the total amount of bacteria was higher in the clean rats in comparison to the contaminated rats, including higher levels of gram-positive bacteria (*Staphylococcus* spp and *Enterococcus* spp) as previously described [1] (Table 1).

**Table 1:** The gut bacterial flora of SOPF rats.

	*Enterococcus spp* /g	*Escherichia coli* /g	*Staphylococcus aureus/g*	*Staphylococcus spp/g*	*Total amount of bacteria/g*
**SOPF**	3100000	< 100	< 100	2800	100000000
**clean**	1500000	< 100	< 100	380	410000000
	920000	< 100	< 100	400	140000000
	5700000	< 100	< 100	< 100	160000000
	110000	< 100	< 100	6200	470000000
*Mean*	2266000	Non detectable	Non detectable	2445	256000000
**SOPF**	140000	36000	280000	< 100	40000000
**contaminated**	160000	1600000	1600000	< 100	42000000
	2000000	44000	320000	< 100	40000000
	240000	460000	140000	< 100	90000000
	3600000	28000	300000	< 100	70000000
*Mean*	1228000	433600	528000	Non detectable	56400000

Values are from SOPF clean (n = 5) and contaminated (n = 5) rats. Mean values are presented for each bacterial strain and the total amount of bacteria from faecal samples.

### Mechanical testing

**Fig 2:**
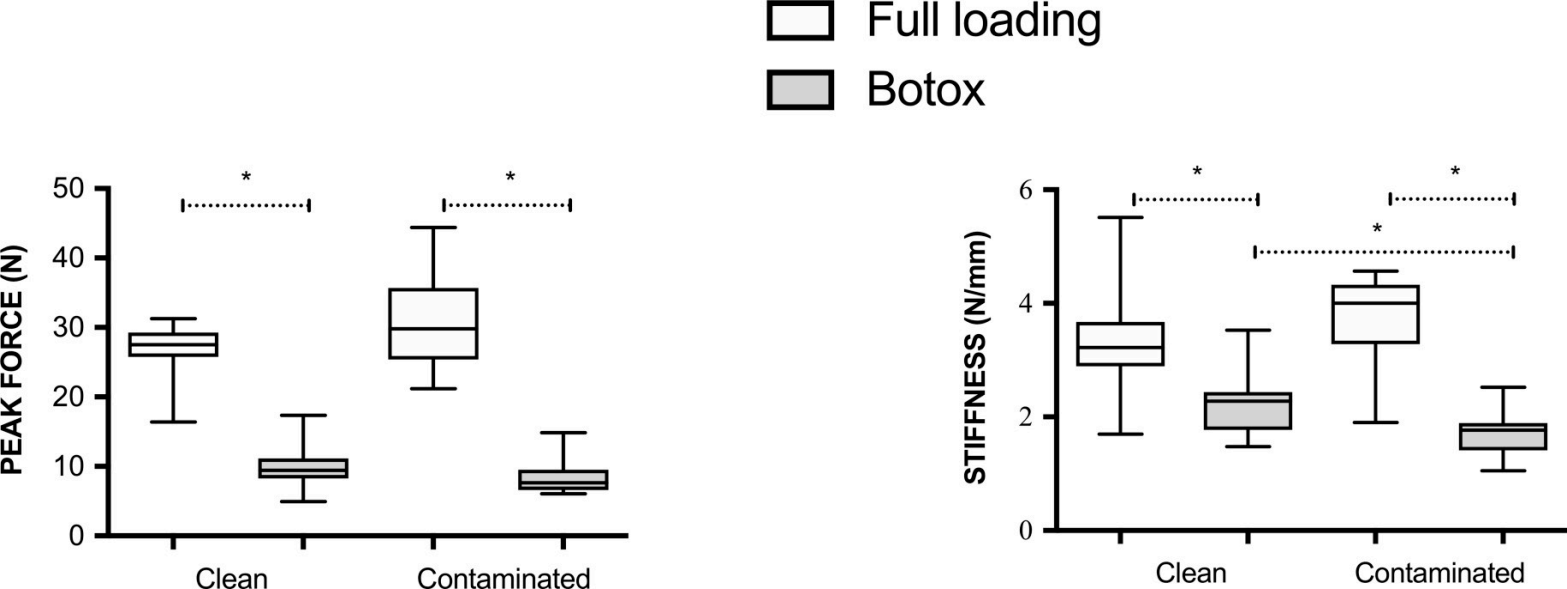
Mechanical data for clean and contaminated rats. Peak force and stiffness at 8 days after tendon transection with or without loading (Botox). Partial unloading by Botox reduced tendon force and stiffness in the clean and contaminated animals. Contamination decreased the stiffness on the partially unloaded animals. Boxes include median, interquartile range and total range (whiskers). (*) Means statistically significant difference (p<0.05).

Partial unloading by Botox had profound effects on most outcome variables (Table 2) and lead to a reduced material and structure properties (Table 3). This was seen in both clean and contaminated rats. The interaction between loading and contamination (by a 2-way ANOVA) was statistically significant for the predetermined primary variable peak force and also for stiffness (p = 0.049 and 0.033 respectively, Table 3). Albeit, looking at the two factors (loading and contamination) individually, it showed that loading had a pronounced response on all variables while contamination had no effect in the 2-way ANOVA. A post hoc analysis showed a small reduction in stiffness (p = 0.042) after contamination in partially unloaded rats, while contamination in fully loaded rats tended to increase the peak force compared to the clean rats (p = 0.056, Table 3 and Fig 2).

**Table 2:** Mechanical results from clean and contaminated rats.

		Full loading X Botox	
		*p*-value	
		Clean	Contaminated
Structural properties	Transverse area (mm^2^)	<0.001	<0.001
	Gap distance (mm)	<0.001	<0.001
	Peak force (N)	<0.001	<0.001
	Stiffness (N/mm)	<0.001	<0.001
	Energy uptake (N/mm)	<0.001	<0.001
Material properties			
	Peak stress (Mpa)	<0.001	<0.001
	Estimate of E-modulus (Mpa)	<0.001	<0.001

**Table 3:** Full loading vs Botox in SOPF animals.

		Clean		Contaminated			Full loading		Botox	
		Full loading	Botox	Full loading	Botox	p-value 2-way ANOVA Interaction	Ratio: Cont/clean	p-value t-test	Ratio: Cont/clean	p-value t-test
		Mean - SD	Mean - SD	Mean - SD	Mean - SD	(Cont-Loading)				
**Structural properties**	Transverse area (mm^2^)	16.80 (4.0)	10.68 (2.4)	17.73 (3.6)	10.83 (2.0)	0.668 (0.563–0.001)	1.06	0.51	1.01	0.88
	Gap distance (mm)	9.90 (0.8)	3.52 (0.5)	10.28 (1.2)	3.96 (0.6)	0.918 (0.097–0.001)	1.04	0.32	1.13	0.06
	Peak force (N)	26.73 (3.7)	9.68 (2.9)	30.7 (6.7)	8.51 (2.9)	0.049 (0.278–0.001)	1.15	0.06	0.87	0.35
	Stiffness (N/mm)	3.35 (0.9)	2.18 (0.5)	3.77 (0.7)	1.71 (0.4)	0.033 (0.908–0.001)	1.12	0.18	0.78	0.04
	Energy uptake (N/mm)	70.26 (15.2)	13.04 (4.4)	72.45 (27.5)	12.62 (5.7)	0.789 (0.856–0.001)	1.03	0.79	0.97	0.85
**Material properties**	Peak stress (Mpa)	1.65 (0.3)	0.95 (0.4)	1.78 (0.4)	0.78 (0.2)	0.149 (0.855–0.001)	1.08	0.37	0.81	0.23
	Estimate of E-modulus (Mpa)	2.00 (0.5)	0.76 (0.3)	2.29 (0.8)	0.64 (0.2)	0.151 (0.571–0.001)	1.15	0.22	0.84	0.27

Clean animals: Full loading (n = 15), Botox (n = 15); Contaminated: Full loading (n = 14), Botox (n = 8). Values are mean and standard deviation (SD).

## Discussion

Our predetermined primary variable was the interaction between loading status and microbiome on tendon callus strength and we found such an effect. There was also an interaction for stiffness. However, the two individual factors showed only a pronounced effect of loading and not by contamination. When analysing separately clean and contaminated animals, loading status alone (full loading vs partial unloading) had a clear effect on all material and structural properties. When microbiome effects were analysed separately, contamination leads to more diverse results depending on the loading status.

In a previous study using the same co-housing design as in this study, changes in the microbiome lead to an altered response to dexamethasone treatment during tendon healing. Furthermore, the controls showed a different response between the clean and contaminated animals where contaminated rats had considerably decreased peak stress and estimate of elastic modulus [1]. One difference in the set-up between our new study and this previous one is the duration of tendon healing. The previous study was evaluated 12 days after tendon transection instead of 8 days after. This could be an explanation to the less pronounced effect of contamination alone in this study.

We have previously used 8 days of healing for several studies on unloaded and loaded healing tendons [6, 7]. However, 8 days is only a few days after the inflammatory phase of healing, which last approximately 3–5 days in rats [11]. To observe a more pronounced response of the microbiota in the mechanical testing we probably need to wait further into the proliferatory and remodelling phase. We have previously seen that there is no mechanical effect 3 days after one single loading episode, however there is a measurable effect 7 days after [10]. Furthermore, new unpublished data shows that loading has the most pronounced effects on tendon healing during 1–2 weeks of healing, but not at 4 weeks (unpublished data). It seems possible that a 12 days healing time in this study could have yielded clearer answers to the secondary outcome variables too.

The strong effect of Botox demonstrates the paramount importance of mechanical loading for tendon healing. This has been shown in numerous previous studies using Botox and other unloading devices in this rat transection model, and does not need further discussion here [6, 11–14]. However, it is worth noticing that the response to contamination tended to diverge between Botox treated and fully loaded rats. The ratio for peak force, between clean and contaminated rats, was 0.87 in the Botox treated animals compared to 1.15 in the fully loaded animals. Previous studies have shown that full loading vs partial unloading activates different mechanisms which ultimately will lead to different mechanical response, more specifically, full loading seems to prolong the proinflammatory response [7].

Our results imply that a change in the rat microbiome using our co-housing method modifies the rat microbiota, as confirmed by both oral swabs tests and faecal analyses. Unpublished data, after only 3 weeks of co-housing, showed that albeit the bacteria had been transferred to the new animals, a longer time was needed to develop a change in the bacterial flora of the animals. This was confirmed by a lack of change in T-cell populations between the clean and contaminated rats in contrast to what we saw after 6 weeks of co-housing [1]. There was also a lack of effect on the peak force. It seems therefore that a longer period of co-housing is needed for the immune system to “mature” upon contact with new bacteria.

Co-housing, for 6 weeks as in this study, will ultimately lead to higher levels of CD3+ and CD4+ T cells in the healing tendon callus [1]. In bone healing, CD4+ T cells are suggested to play a beneficial role in tissue regeneration [15]. This might explain the tendency to stronger tendons in fully loaded contaminated rats compared to the clean (p = 0.056). The early callus is dominated by different leukocytes, and the composition of these cells undergoes dramatic change during the early healing phases in our model [8–10]. Full loading, in contrast to partial unloading, can result in tissue microdamage followed by a pro-inflammatory response [7]. As full loading, but not partial unloading, creates microdamage, it could explain the divergent response to contamination in with different degrees of loading.

Immunomodulatory changes by the microbiome have probably more profound effects on the healing of fully loaded tendons. The Botox model for partial unloading is however probably more similar to the clinical situation after an Achilles tendon rupture where the patients have the lower leg immobilized in an orthosis or cast. The response to small loads in Botox treated animals probably acts primarily through mechanotransduction while the response in fully loaded animals acts probably through a combination of a mechanotransduction and microdamage. Partial unloading by Botox can also change the composition of T cells in the tendon callus [9] but when we compare partial unloaded clean and contaminated rats, contamination lead to a significant decrease in the stiffness of the healing tendon. However, a limitation with this study is that the fully loaded animals did not get a sham injection in the calf muscle, but we believe that it is unlikely that this minor muscle needling itself would influence the tendon healing.

Another limitation of the study is that the mechanisms of how the gut microbiota influence tendon healing was not elucidate. We only used a standard commercial test for faecal analysis in this study and no extensive bacteriological analysis was performed. This specific test was chosen to confirm a change in the microbiota, and not to verify which strain of bacteria that are important for tendon healing. The objectives with this study was to understand if the response to different loading conditions were affected by the microbiome and albeit the effects are minor, we did confirm a link between microbiome changes and the loading status during tendon healing.

Taken together, the microbiome influences tendon healing and the response to mechanical loading. The use of different levels of clean animals could possibly lead to difficulties in reproducing results. Future experiments aiming for clinical relevance should probably take the microbiome into account when studying the response to loading in animal models.

## Conclusion

The gut microflora has an effect on mechanical loading for tendon healing stimulation.

## Materials and methods

### Experimental design

**Fig 3:**
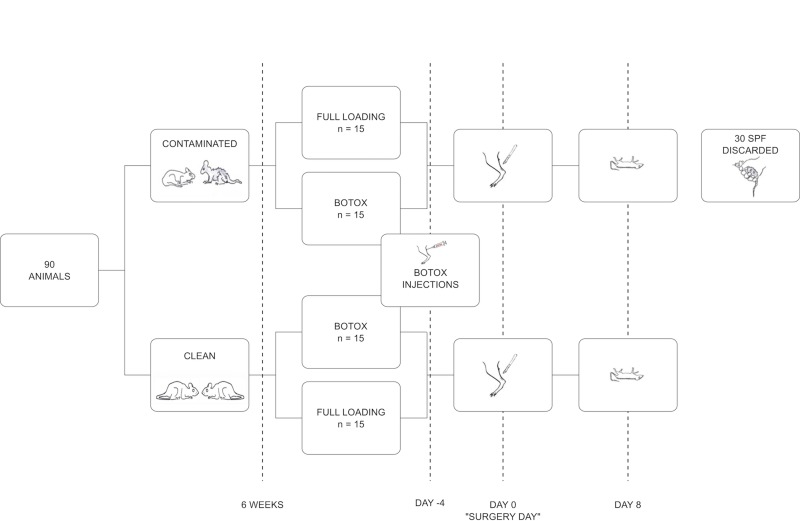
Experimental set-up.

In total 90 rats were used for this study: 60 specific opportunist and pathogen free (SOPF) rats and 30 specific pathogen free (SPF). The SPF rats were bred under less clean conditions and were known to carry *Staphylococcus aureus*. 30 of the SOPF rats were co-housed one by one with an SPF rat for 6 weeks for contamination by microorganisms carried by the SPF rats. The remaining SOPF rats were kept in a separate part of the department to ensure that they were remaining clean but were also housed in pairs. Only SOPF rats (clean and contaminated) were used for data collection. These animals were randomized to either full loading or partial unloading (mild loading) before the contamination process was performed (Fig 3). Partial unloading was achieved by Botox injections in the calf muscles. Thereafter, all rats underwent Achilles tendon transection and eight days later, tendons were harvested and mechanically tested. Our pre-determined primary outcome variable was an interaction between loading and contamination for peak force, measured by a 2-way ANOVA.

#### Animals and housing

Female SOPF and SPF Sprague-Dawley rats, weighing on average of 275 grams (SD 23), 11 to 12 weeks old, were purchased from Janvier, Le Genest-Saint-Isle, France, from two different breeding facilities. All experiments were approved by the local ethical committee (Regional Ethics Committee for Animal Experiments in Linköping, ref. 15–15 and 592). The experiments were adhered to institutional guidelines for care and treatment of laboratory animals, and all efforts were made to minimize suffering. All animals were housed in acrylic cages, two by two, and placed on ventilated racks with humidity (55%), temperature (22°C) and light-dark cycle (12h each). Standard food pellets and water was given *ad libitum*.

#### Co-housing model and contamination procedure

The SOPF rats were, according to the breeder, free of *Bordetella bronchiseptica*, *CAR bacillus*, *Clostridium piliforme (tyzzer)*, *Corynebacterium kutscheri*, *Dermatophytes*, *Encephalitozoon cuniculi*, *Helicobacter spp*, *Klebsiella oxytoca/pneumoniae*, *Mycoplasma pulmonis*, *Pasteurellaceae sp*., *Pneumocystis spp*, *Proteus spp (mirabilis*, *vulgaris)*, *Pseudomonas aeruginosa*, *Salmonella spp*., *Staphylococcus aureus*, *Streptobacillus moniliformis*, *Streptococci ß-hemolytic*, and *Streptococcus pneumoniae*. While all SPF rats carried *Staphylococcus aureus* and *Pseudomonas aeruginosa* at arrival. The *Staphylococcus aureus* was confirmed by oral swab test and bacterial culture as this was the intended bacterial strain that we wanted to contaminate the SOPF rats with. Contamination was performed by co-housing one SOPF rat with one SPF rat, as described previously [1, 16]. After 6 weeks of co-housing, contamination was confirmed in the SOPF rats by testing for *Staphylococcus aureus* by an oral swab test and bacterial culture. Bacteria were grown on CHROMagar Staph Chrom plates for 24 hours and the result was graded as 0, 1, 2 or 3, where 0 means no detected *Staphyloccocus aureus*, 1 means minimal, 2 more than half, and 3 dominant presence. Only rats with growth grade 2 and 3 were used for analysis.

#### Botox injections for partial unloading

30 rats (15 clean and 15 contaminated) were anesthetized with isoflurane gas followed by botulinum toxin (Botox, Allergan, Irvine, CA) in the right hind leg. The injections were performed into the gastrocnemius lateralis, medialis and soleus muscles (dose of 1 U/muscle, in total 3 U/animal and a volume of 0.06 mL). The animals were thereafter allowed free cage activity. Botox effectiveness was confirmed prior to the surgery by visual inspection.

#### Tendon transection

Four days later, both the partial unloaded and the fully loaded rats were anesthetized with isoflurane gas and the Achilles tendon, from the right hind leg was transected. To avoid postoperative infection, antibiotic was given once preoperatively (25 mg/kg oxytetracycline). Analgesic was given subcutaneously pre- and postoperatively every 8-12h for 48h (0.045 mg/kg buprenorphine). Surgery was performed under aseptic conditions and the skin on the right lower leg was shaved and cleaned with chlorhexidine ethanol. The Achilles and plantaris tendon complex was exposed through a transverse skin incision on the lateral side. The plantaris tendon was removed completely and the Achilles tendon was cut transversely in the middle part and was left unsutured whereas the skin was closed with 2 stitches. This procedure has been described elsewhere [3].

#### Mechanical testing

Eight days after surgery, the rats were anesthetized with isoflurane gas and killed with carbon dioxide. The right Achilles tendon was harvested together with the calcaneal bone and calf muscle. Sagittal and transverse diameter of the midpart of the callus tissue was measured with a slide calliper, and the transverse area was calculated by assuming an elliptical geometry. The distance between the old tendon stumps (gap distance) was measured, as seen through the partly transparent callus tissue. The muscle tissue was scraped off from the tendon, which was fixed in a metal clamp with sandpaper. The bone was fixed in a custom-made clamp at 30° dorsiflexion relative to the direction of traction in the materials-testing machine (100R; DDL, Eden Prairie, MN). The machine pulled at 0.1 mm/s until failure. Peak force at failure (N) and energy uptake (N/mm), were calculated by the software of the machine. The investigator marked a linear portion of the elastic phase of the curve for automated stiffness (N/mm) calculation. Peak stress (MPa) and estimate of elastic modulus (MPa) were calculated assuming an elliptical cylindrical shape and homogeneous mechanical properties. Estimation of elastic modulus was calculated as stiffness*gap distance/transverse area [1, 3].

#### Characterization of gut bacterial flora

After the tendon samples were collected, SOPF rats (5 clean and 5 contaminated) from the full loading group were used to confirm changes in the microbial community. Faecal pellets were collected via laparotomy under aseptic conditions. The microbial community was identified by Surrey Diagnostics Ltd using the API (BioMerieux) and RapID (Remel) biochemical test. These data have previously been described as they were not collected solely for the present study [1].

#### Statistics

The mechanical results were analysed using SPSS software version 21. Only SOPF animals (clean and contaminated) were included. SPF rats were discarded. Our predefined primary outcome was the interaction between microbiome (contamination) and loading status for peak force, measured by a 2-way ANOVA. For post hoc, we compared groups pairwise using Student’s t-test to describe the response to contamination among loaded and partial unloading rats separately. The presence of *Staphyloccocus aureus* and the tests of bacteriological status in the gut microbiota were only descriptive.

## Supporting information

S1 Dataset Data from mechanical evaluation and the status of the gut bacterial flora. Data are from each rat.
(XLSX)

## References

[1] Dietrich-Zagonel F, Hammerman M, Tatting L, Dietrich F, Kozak Ljunggren M, Blomgran P, et al Stimulation of Tendon Healing With Delayed Dexamethasone Treatment Is Modified by the Microbiome. The American journal of sports medicine. 2018;46(13):3281–7. 10.1177/0363546518799442 30265844

[2] Blomgran P, Hammerman M, Aspenberg P. Systemic corticosteroids improve tendon healing when given after the early inflammatory phase. Scientific reports. 2017;7(1):12468 10.1038/s41598-017-12657-0 28963482 PMC5622078

[3] Dietrich F, Hammerman M, Blomgran P, Tatting L, Bampi VF, Silva JB, et al Effect of platelet-rich plasma on rat Achilles tendon healing is related to microbiota. Acta orthopaedica. 2017;88(4):416–21. 10.1080/17453674.2017.1293447 28296518 PMC5499334

[4] Linehan JL, Harrison OJ, Han SJ, Byrd AL, Vujkovic-Cvijin I, Villarino AV, et al Non-classical Immunity Controls Microbiota Impact on Skin Immunity and Tissue Repair. Cell. 2018;172(4):784–96 e18. 10.1016/j.cell.2017.12.033 PMC6034182 29358051

[5] Furusawa Y, Obata Y, Fukuda S, Endo TA, Nakato G, Takahashi D, et al Commensal microbe-derived butyrate induces the differentiation of colonic regulatory T cells. Nature. 2013;504(7480):446–50. 10.1038/nature12721 24226770

[6] Andersson T, Eliasson P, Hammerman M, Sandberg O, Aspenberg P. Low-level mechanical stimulation is sufficient to improve tendon healing in rats. J Appl Physiol (1985). 2012;113(9):1398–402. 22936727 10.1152/japplphysiol.00491.2012

[7] Hammerman M, Dietrich-Zagonel F, Blomgran P, Eliasson P, Aspenberg P. Different mechanisms activated by mild versus strong loading in rat Achilles tendon healing. PloS one. 2018;13(7):e0201211 10.1371/journal.pone.0201211 30044869 PMC6059492

[8] Hammerman M, Blomgran P, Dansac A, Eliasson P, Aspenberg P. Different gene response to mechanical loading during early and late phases of rat Achilles tendon healing. Journal of applied physiology. 2017;123(4):800–15. 10.1152/japplphysiol.00323.2017 28705996

[9] Blomgran P, Blomgran R, Ernerudh J, Aspenberg P. A possible link between loading, inflammation and healing: Immune cell populations during tendon healing in the rat. Scientific reports. 2016;6:29824 10.1038/srep29824 27405922 PMC4942825

[10] Eliasson P, Andersson T, Aspenberg P. Influence of a single loading episode on gene expression in healing rat Achilles tendons. Journal of applied physiology. 2012;112(2):279–88. 10.1152/japplphysiol.00858.2011 21998267

[11] Andersson T, Eliasson P, Aspenberg P. Tissue memory in healing tendons: short loading episodes stimulate healing. Journal of applied physiology. 2009;107(2):417–21. 10.1152/japplphysiol.00414.2009 19541735

[12] Eliasson P, Andersson T, Aspenberg P. Achilles tendon healing in rats is improved by intermittent mechanical loading during the inflammatory phase. Journal of orthopaedic research: official publication of the Orthopaedic Research Society. 2012;30(2):274–9. 21809382 10.1002/jor.21511

[13] Eliasson P, Andersson T, Aspenberg P. Rat Achilles tendon healing: mechanical loading and gene expression. Journal of applied physiology. 2009;107(2):399–407. 10.1152/japplphysiol.91563.2008 19541731

[14] Freedman BR, Gordon JA, Bhatt PR, Pardes AM, Thomas SJ, Sarver JJ, et al Nonsurgical treatment and early return to activity leads to improved Achilles tendon fatigue mechanics and functional outcomes during early healing in an animal model. Journal of orthopaedic research: official publication of the Orthopaedic Research Society. 2016;34(12):2172–80. 27038306 10.1002/jor.23253 PMC5047851

[15] Schlundt C, Schell H, Goodman SB, Vunjak-Novakovic G, Duda GN, Schmidt-Bleek K. Immune modulation as a therapeutic strategy in bone regeneration. Journal of experimental orthopaedics. 2015;2(1):1 10.1186/s40634-014-0017-6 26914869 PMC4545842

[16] Beura LK, Hamilton SE, Bi K, Schenkel JM, Odumade OA, Casey KA, et al Normalizing the environment recapitulates adult human immune traits in laboratory mice. Nature. 2016;532(7600):512–6. 10.1038/nature17655 27096360 PMC4871315

